# P-309. Spread of SCC*mec* IV type Community-acquired MRSA in a Tertiary Care ICU in Japan

**DOI:** 10.1093/ofid/ofae631.512

**Published:** 2025-01-29

**Authors:** Hideki Kawamura, Daisuke Okawa, Syuhei Niiyama, Naoko Imuta, Junichiro Nishi, Yasuyuki Kakihana

**Affiliations:** Kagoshima University, Kagoshima, Kagoshima, Japan; Kagoshima University Hospital, Kagoshima-city, Kagoshima, Japan; Kagoshima University Hospital, Kagoshima-city, Kagoshima, Japan; Kagoshima University Graduate School of Medicine and Dental Sciences, Kagoshima-city, Kagoshima, Japan; Kagoshima University Hospital, Kagoshima-city, Kagoshima, Japan; Kagoshima University Hospital, Kagoshima-city, Kagoshima, Japan

## Abstract

**Background:**

In recent years, community-acquired (CA) MRSA has become a problem, but there have been few reports of MRSA genotypes in the ICU. In our ICU, we have reinforced countermeasures such as screening for MRSA carriers using nasal specimens at admission and every week thereafter and decolonizing those who test positive for MRSA using mupirocin. This study aimed to investigate the prevalent strains of MRSA in the intensive care setting and their risk based on the genotyping of MRSA strains from patients admitted to our ICU.

**Figure 1.**
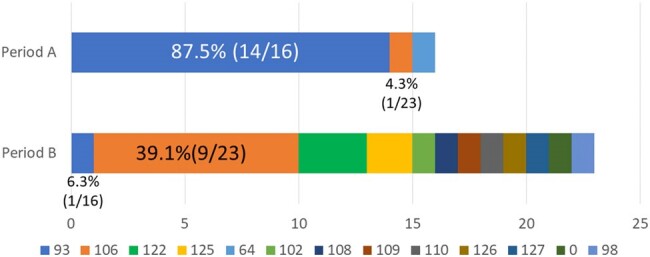
Comparison of the number of detections by POT1 value between Period A and Period B

Strains in Period A had more NY/Japan clones with a POT1 value of 93, while those in Period B had more community-acquired strains with a POT1 value of 106.

**Methods:**

We examined two periods of increased nosocomial MRSA infections: 2013 to 2014 (16 strains in Period A) and 2021 to 2022 (23 strains in Period B). We evaluated changes in epidemic strains and assessed the possibility of transmission from ICU patients by genotyping of MRSA strains using PCR-based ORF Typing (POT).

**Figure 2.**
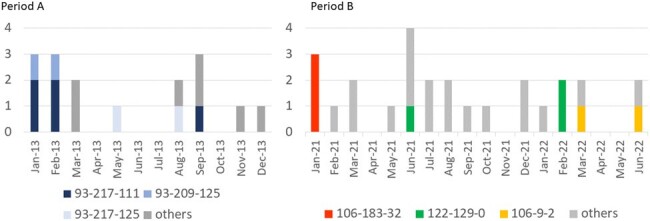
Epidemic curves by genotype in Period A and in Period B

There were three cases in Period A and two in Period B with the same genotype strain in hospitalized patients during the same period, all of which in Period A showed a POT1 value of 93.

**Results:**

In Period A, 87.5% (14/16) of the strains showed a POT1 value of 93, presumed to be the NY/Japan clone; however, in Period B, the number of strains showing a POT1 value of 93 decreased to 4.3% (1/23) (p < 0.001). In period A, 6.3% (1/16) of the strains showed a POT1 value of 106, presumed to be a CA strain. However, this increased to 39.1% (9/23) in period B (p = 0.01), and these strains were considered epidemic (Figure 1). There were no PVL-positive strains in Period A and Period B. There were three cases in Period A and two in Period B with the same genotype strain in hospitalized patients during the same period, all of which in Period A showed a POT1 value of 93 (Figure 2). Four cases (25.0%; three cases of pneumonia with bacteremia and one of surgical site infection) in Period A and 8 cases (34.8%; 6 cases of pneumonia (including 3 cases of bacteremia) and 2 cases of central line-associated bloodstream infections) in Period B developed infections.

**Conclusion:**

The results suggest that CA MRSA strains are being brought into the ICU and causing infections, although the number of conventional nosocomial strains has decreased due to enhanced countermeasures. Monitoring of CA MRSA in ICUs is needed.

**Disclosures:**

**All Authors**: No reported disclosures

